# Quality of Life After Surgery or Surveillance for Asymptomatic Primary Hyperparathyroidism: A Meta-Analysis of Randomized Controlled Trials

**DOI:** 10.1097/MD.0000000000000931

**Published:** 2015-06-12

**Authors:** Shih-Ping Cheng, Jie-Jen Lee, Tsang-Pai Liu, Po-Sheng Yang, Sung-Chen Liu, Yi-Chiung Hsu, Chien-Liang Liu

**Affiliations:** From the Department of Surgery, MacKay Memorial Hospital and Mackay Medical College (S-PC, J-JL, T-PL, P-SY, C-LL); Division of Endocrinology and Metabolism, Department of Medicine, MacKay Memorial Hospital and Mackay Medical College (S-CL); Mackay Junior College of Medicine, Nursing, and Management (J-JL, T-PL, S-CL, C-LL); Department of Pharmacology and Graduate Institute of Medical Sciences, Taipei Medical University (S-PC, J-JL); and Institute of Statistical Science, Academia Sinica, Taipei, Taiwan (Y-CH).

## Abstract

A number of studies have investigated the effects of surgery on symptoms and quality of life in patients with hyperparathyroidism. However, the results are inconsistent. We conducted this meta-analysis to quantitatively assess changes in quality of life among patients with asymptomatic primary hyperparathyroidism.

Different databases were searched for randomized controlled trials comparing surgery with surveillance. Quality of life was measured by the Short Form-36 general health survey. The pooled random-effects estimates of standardized mean difference (SMD) and 95% confidence intervals (CIs) were calculated.

Three trials involving 294 participants were included. At 1 year, patients undergoing parathyroidectomy had significantly better physical role functioning (SMD, 0.31; 95% CI 0.04–0.57; *P* = 0.02) and emotional role functioning (SMD, 0.29; 95% CI 0.02–0.55; *P* = 0.03). At 2 years, the surgery group had significantly better emotional role functioning (SMD, 0.35; 95% CI 0.02–0.67; *P* = 0.04) than the surveillance group. Furthermore, compared with baseline, emotional role functioning improved after surgery (SMD, 0.31; 95% CI 0.02–0.60; *P* = 0.04), whereas emotional role functioning tended to get worse in patients assigned to medical surveillance (SMD, −0.27; 95% CI −0.55 to 0.02; *P* = 0.07).

Although Short Form-36 is a generic instrument, our results suggest that parathyroidectomy may be associated with better quality of life, especially in the emotional aspects of well-being.

## INTRODUCTION

Classic symptoms and signs of primary hyperparathyroidism are uncommon nowadays. With the addition of calcium to routine biochemical screening in the 1970s, the incidence of primary hyperparathyroidism has substantially increased, along with the discovery of many asymptomatic cases. Depending on the length of follow-up, up to 37% of asymptomatic patients who do not undergo surgery showed disease progression.^1–3^ Patients with untreated hyperparathyroidism may have stable bone mineral density (BMD) or slow loss in the long run.^4^ Conversely, asymptomatic patients have stable increases in BMD after surgery. Although randomized controlled trials suggested renal function remained stable and did not differ between medical surveillance and surgery,^5–7^ a record-linkage cohort study demonstrated a markedly increased risk of nephrolithiasis during medical surveillance of patients with asymptomatic hyperparathyroidism.^8^ These benefits are reflected in the surgical indications recommended by all the versions of guidelines from the International Workshop.^9^

Patients with asymptomatic hyperparathyroidism also exhibit neuropsychiatric and cognitive changes, which may have an impact on quality of life. Patient-centered outcomes, including symptoms and quality of life, are as important as clinical outcomes such as mortality and morbidity.^10^ Two measures have been used widely in hyperparathyroidism: the generic Short Form-36 (SF-36) general health survey and the specific parathyroidectomy assessment of symptoms score.^11^ It has been shown that patients undergoing parathyroidectomy had improved symptoms and quality of life comparable with the thyroidectomy group at 10 years.^12^ Furthermore, the 2 measures may correlate with each other.^13^ A number of studies have demonstrated improvement in either measure after parathyroidectomy.^14^

An earlier systematic review found that improvements in energy level and both physical and emotional well-being were the most commonly reported postoperative findings.^15^ Recently, the consensus statements from the Fourth International Workshop on primary hyperparathyroidism indicated that specific findings of 3 randomized controlled studies were inconsistent.^16^ Therefore, neurocognitive elements were not included in the recommendations for surgery. There is a need to systematically review the relevant studies to guide future research and recommendations. The objective of this study was to quantitatively assess changes in quality of life among patients with asymptomatic primary hyperparathyroidism.

## METHODS

### Search Strategy

The meta-analysis was carried out in accordance with the Preferred Reporting Items for Systematic Reviews and Meta-Analyses guidelines.^17^ A literature search was performed using the terms “primary hyperparathyroidism” and “quality of life” or “SF-36.” The following databases were searched in December 2014: Cochrane Collaboration Database, MEDLINE, EMBASE, and Cumulative Index to Nursing and Allied Health Literature. Studies were included regardless of language or publication status. Additional studies in the reference lists of the retrieved articles, including relevant meta-analyses and systematic reviews, were also searched. The approval by an institutional review board is not required because this study was based on published trials.

### Selection Criteria

We included randomized controlled trials comparing surgery with surveillance in patients with asymptomatic (mild) primary hyperparathyroidism. Included studies had to report quantitative analysis of quality of life.

### Data Extraction

Each of the potentially eligible studies was independently assessed by 2 of the authors using a predesigned data extraction table. The name of the first author and the year of publication of the article were used for identification. The patients’ quality of life was measured with the SF-36 general health survey. The self-administered SF-36 survey consists of 36 questions that evaluate 8 discrete domains: physical functioning, physical role functioning, bodily pain, general health, vitality, social functioning, emotional role functioning, and mental health.^18^ The analyses were performed on data at baseline, 6-month, 1-, and 2-year time points. Only 1 study reported data at time points beyond 2 years.^5^ If data were not available from the text or tables, they were measured from the published figures.

### Quality Assessment

Study methodology and risk of bias were evaluated by considering randomization procedure, allocation concealment, blinding, and follow-up. The quality of reporting was assessed by the Jadad score, which determines quality of a clinical trial using 3 items (blinding, randomization, and description of withdrawals and dropouts).^19^ The range of possible Jadad scores is 0 (poor) to 5 (good). Funnel plots were used to examine the possibility of publication bias.^20^ In the absence of bias, the plot will resemble a symmetrical inverted funnel.

### Statistical Analysis

A pairwise meta-analysis for studies that compared quality of life between the 2 groups at different time points was performed. We also performed meta-analysis for the each group comparing quality of life at 6, 12, and 24 months with that at baseline. Statistical analysis was performed using STATA 12.0 (Stata Corp, College Station, TX). Estimated effect sizes were expressed as standardized mean difference (SMD) and 95% confidence intervals (CIs) with a correction factor (Hedges’ *g*). We evaluated potential heterogeneity across studies using a χ^2^ test and the *I*^2^ statistic. All meta-analyses were performed using the random effects model due to the variability in the study populations. A *P* value of < 0.05 was considered statistically significant.

## RESULTS

### Study Characteristics

**FIGURE 1:**
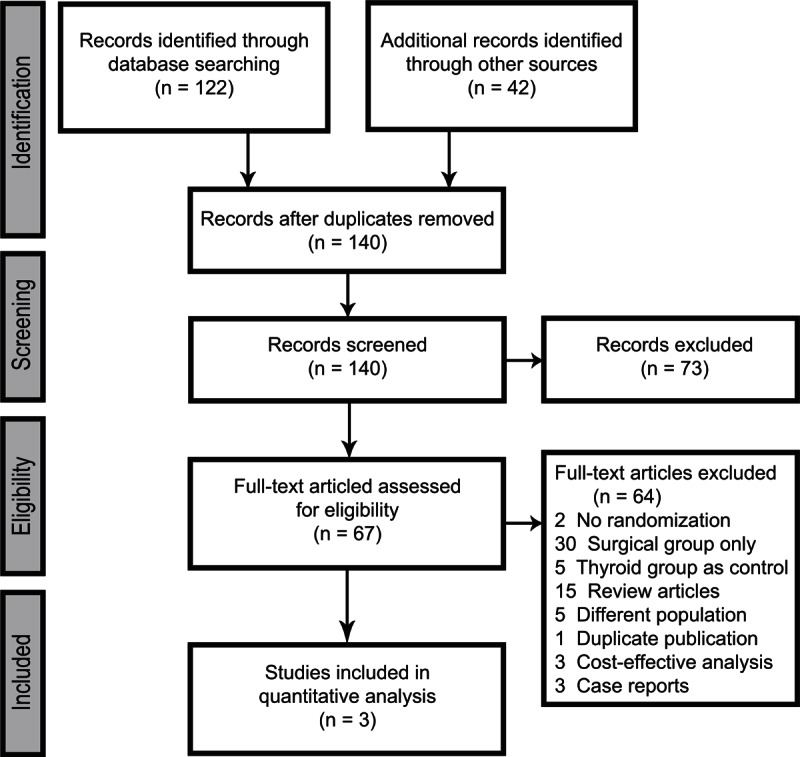
Preferred Reporting Items for Systematic Reviews and Meta-Analyses diagram summarizing literature screening process.

Electronic and reference searches recovered 164 publications (Figure 1) and only 3 randomized controlled studies met our criteria for the meta-analysis. Two studies showed the same number and period of patient enrollment.^5,21^ Only the study in which the exact SF-36 scores were provided was included for analysis. Finally, a total of 145 patients underwent parathyroidectomy, whereas 149 patients had medical surveillance. Table 1 shows the characteristics of the studies.

**TABLE 1:**
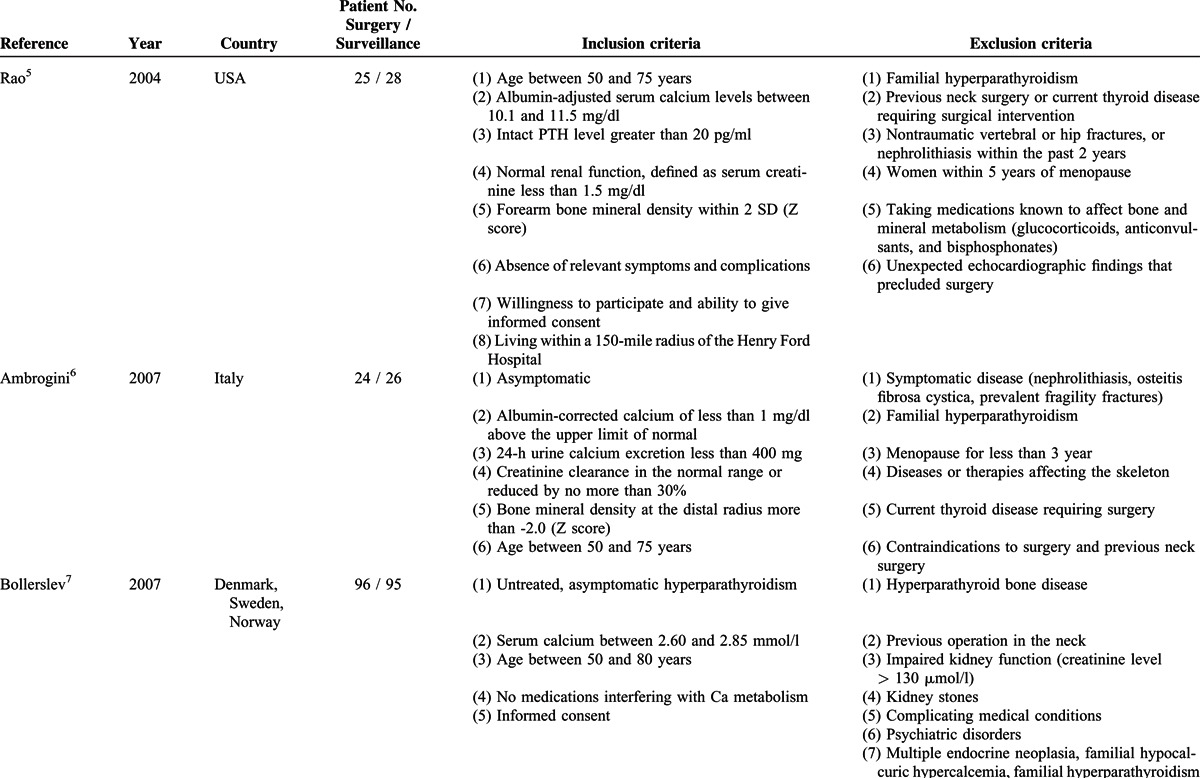
Characteristics of included randomized controlled trials

All included trials reported scores on the 8 domains of the SF-36 as measures of quality of life. In all studies, the scores had to be extracted from figures. Two studies reported scores at 6 months,^5,6^ 3 reported scores at 12 months,^5–7^ and 2 reported scores at 24 months.^5,7^ Changes in BMD were also evaluated in these studies and have been analyzed previously.^4^

Table 2 summarizes the quality assessment of the included studies. Lack of blinding may be the inherent limitations in all trials.

**TABLE 2:**
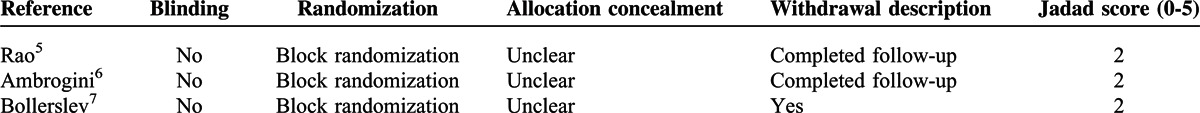
Quality of included randomized controlled trials

### Primary Outcome Analysis

At baseline, there was no significant difference across all domains of the SF-36 between the surveillance group and surgery group (Figure 2   A). There was no heterogeneity among studies except emotional role functioning (*I*^2^ = 73.7%).

**FIGURE 2:**
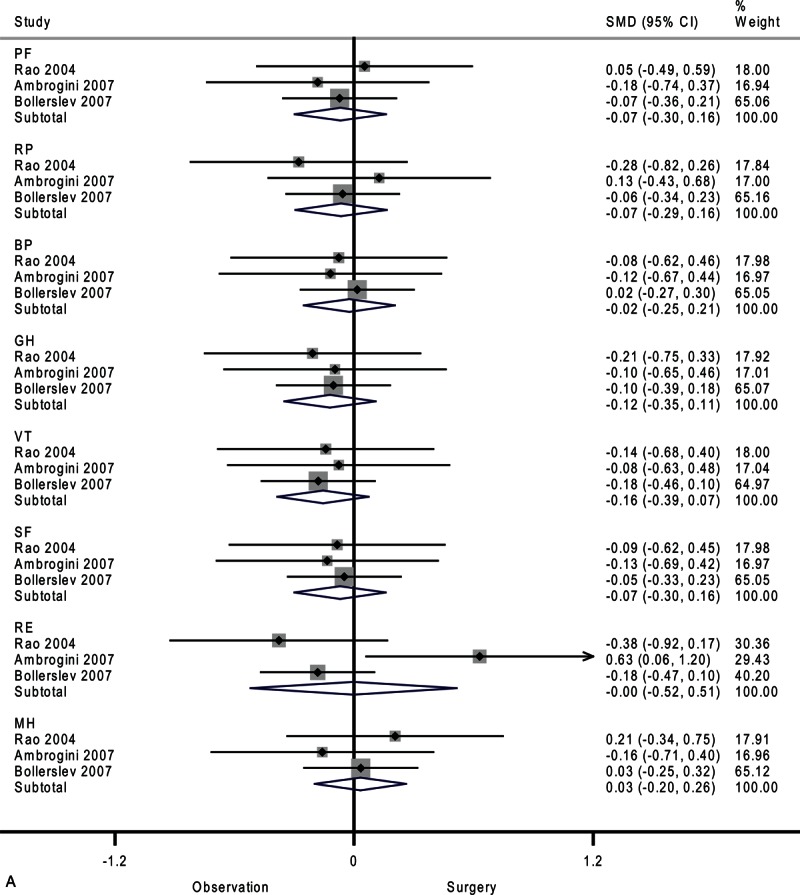
Forest plots showing standardized mean difference and 95% confidence intervals of the Short Form-36 general health survey between surgery and surveillance groups at baseline (A), 6 months (B), 12 months (C), and 24 months (D). The diamonds represent the overall pooled estimate.

**FIGURE 2 (Continued):**
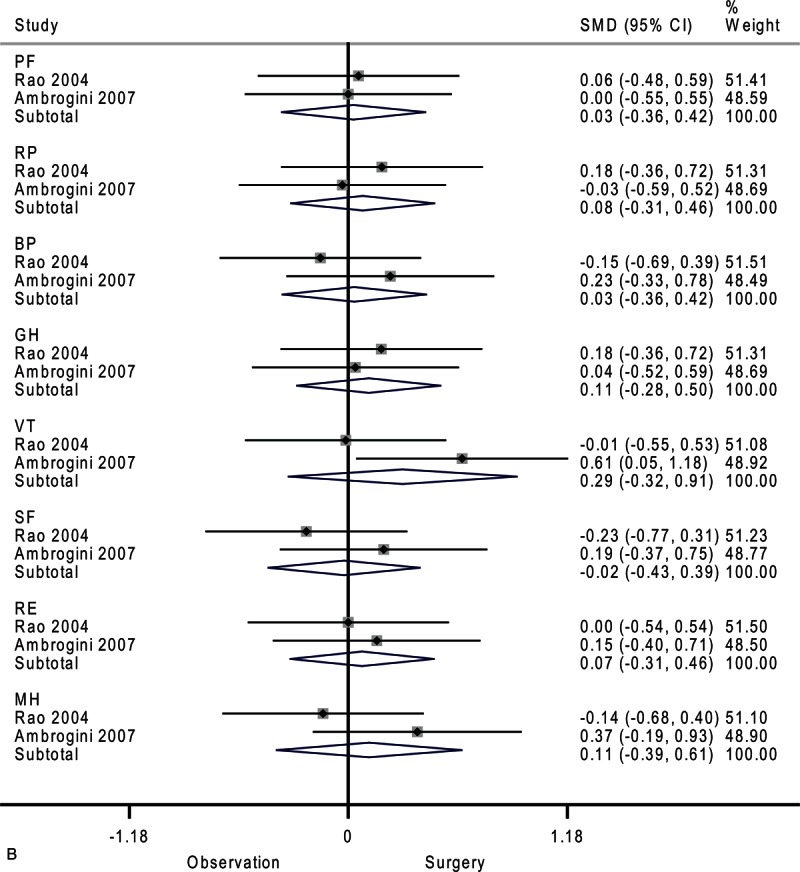
Forest plots showing standardized mean difference and 95% confidence intervals of the Short Form-36 general health survey between surgery and surveillance groups at baseline (A), 6 months (B), 12 months (C), and 24 months (D). The diamonds represent the overall pooled estimate.

**FIGURE 2 (Continued):**
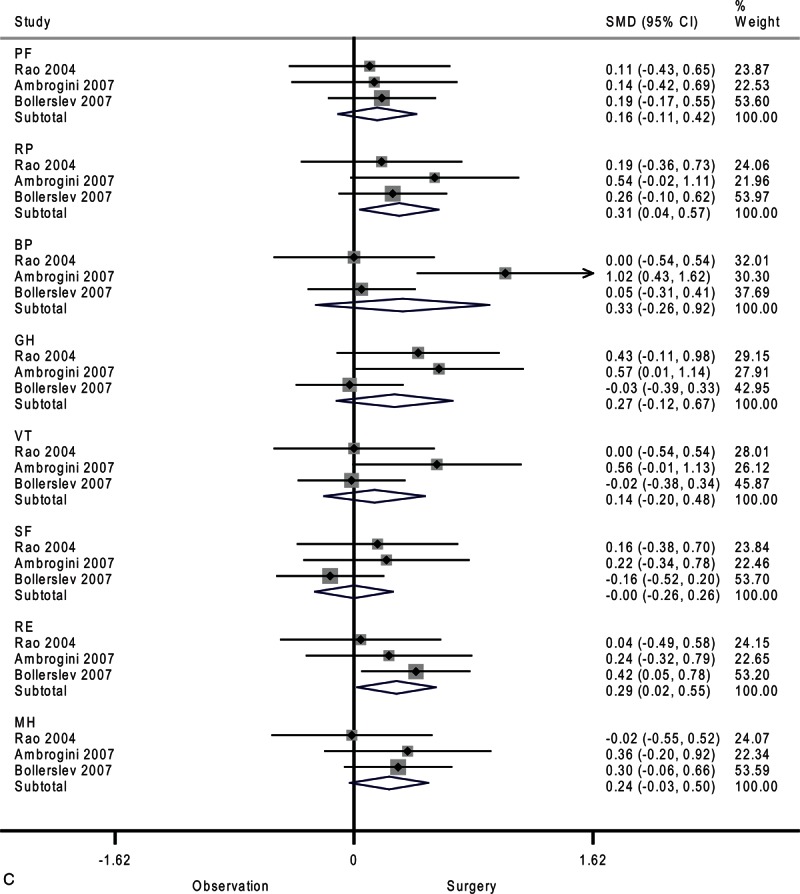
Forest plots showing standardized mean difference and 95% confidence intervals of the Short Form-36 general health survey between surgery and surveillance groups at baseline (A), 6 months (B), 12 months (C), and 24 months (D). The diamonds represent the overall pooled estimate.

**FIGURE 2 (Continued):**
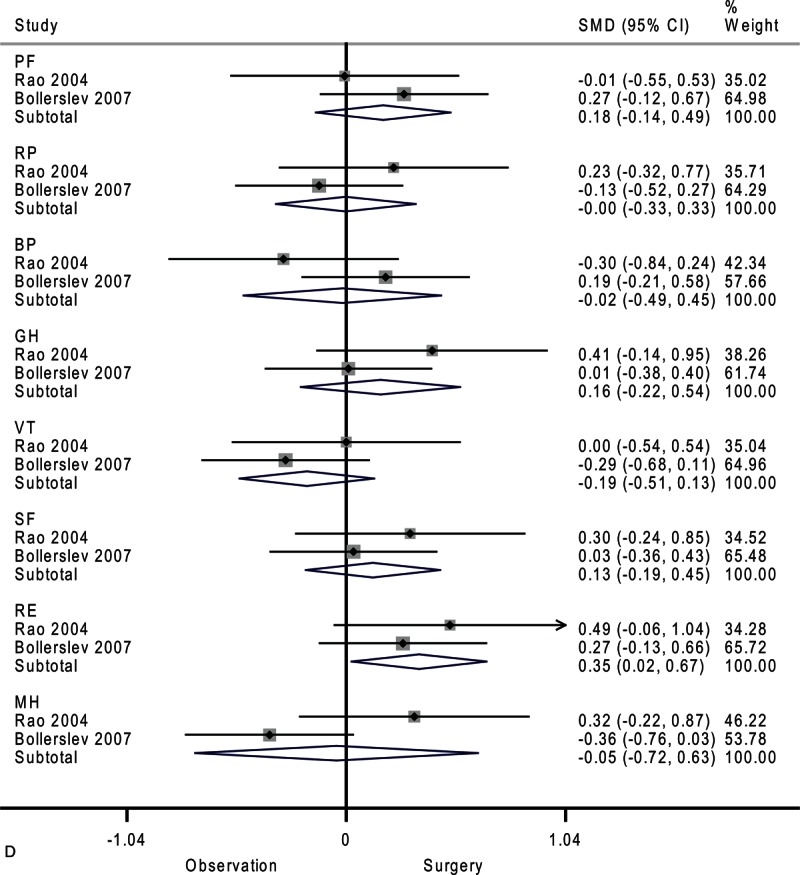
Forest plots showing standardized mean difference and 95% confidence intervals of the Short Form-36 general health survey between surgery and surveillance groups at baseline (A), 6 months (B), 12 months (C), and 24 months (D). The diamonds represent the overall pooled estimate.

After 6 months, the surveillance group and surgery group showed similar SF-36 scores (Figure 2   B). Moderate heterogeneity among studies was observed in vitality (*I*^2^ = 59.5%) and mental health (*I*^2^ = 39.3%).

**FIGURE 3:**
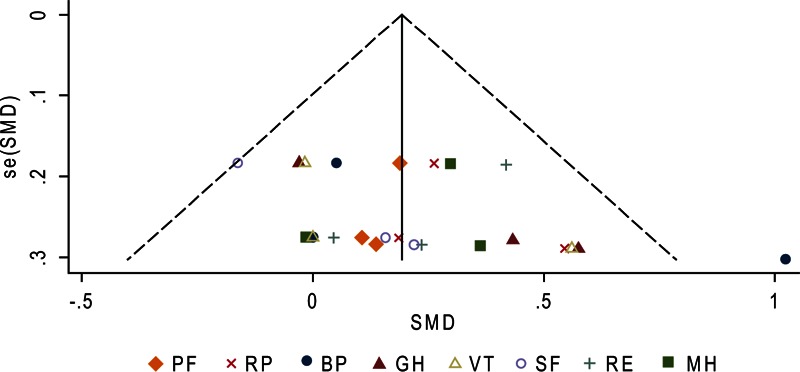
Funnel plot to explore potential publication bias for the Short Form-36 (SF-36) general health survey between surgery and surveillance groups at 12 months. Effect estimates of individual studies (standardized mean difference [SMD]) are scattered against the precision of the studies (standard error of SMD).

As shown in Figure 2      C, after 12 months, the surgery group showed better physical role functioning (SMD, 0.31; 95% CI 0.04–0.57; *P* = 0.02) and emotional role functioning (SMD, 0.29; 95% CI 0.02–0.55; *P* = 0.03). The heterogeneity among studies was high in bodily pain (*I*^2^ = 76.5%) and moderate in general health (*I*^2^ = 49.2%) and vitality (*I*^2^ = 34.8%). A funnel plot of study size against treatment effect for the 3 included trials showed minimal asymmetry, which did not suggest publication bias (Figure 3).

After 24 months, only emotional role functioning remained significantly different between the surveillance group and surgery group as shown in Figure 2      D (SMD, 0.35; 95% CI 0.02–0.67; *P* = 0.04). The heterogeneity among studies was high in mental health (*I*^2^ = 75.0%) and moderate in bodily pain (*I*^2^ = 50.1%) and general health (*I*^2^ = 25.2%).

### Secondary Outcome Analysis

Next, we analyzed temporal changes within each group. Compared with baseline, the surveillance group had no significant change in quality of life after 6 months (Figure 4     A). However, physical role functioning was worse after 12 months (SMD, −0.37; 95% CI −0.62 to −0.12; *P* = 0.003). As shown in Figure 4     , there was a trend toward lower scores on 3 domains of the SF-36: bodily pain (SMD, −0.28; 95% CI −0.59 to 0.03; *P* = 0.07), general health (SMD, −0.23; 95% CI −0.48 to 0.01; *P* = 0.06), and social functioning (SMD, −0.22; 95% CI −0.47 to 0.03; *P* = 0.08). Moderate-to-substantial heterogeneity among studies was observed in emotional role functioning (*I*^2^ = 62.1%).

**FIGURE 4:**
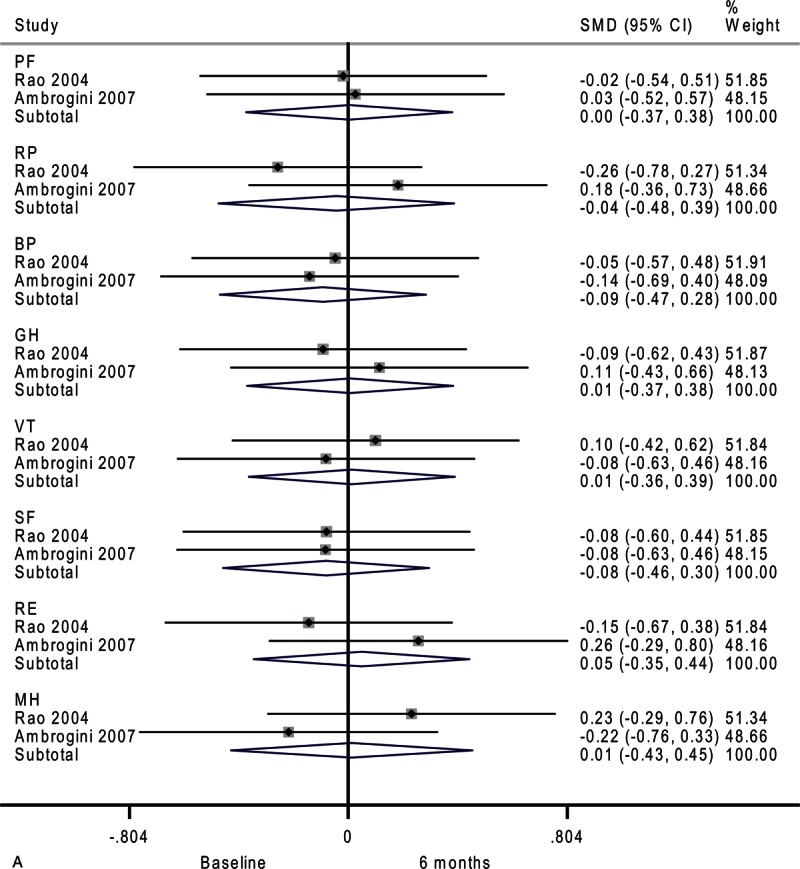
Forest plots showing standardized mean difference (SMD) and 95% confidence intervals (CIs) of the Short Form-36 (SF-36) general health survey in surveillance (A, C, E) and surgery (B, D, F) groups at 6 months (A, B), 12 months (C, D), and 24 months (E, F). The diamonds represent the overall pooled estimate.

**FIGURE 4 (Continued):**
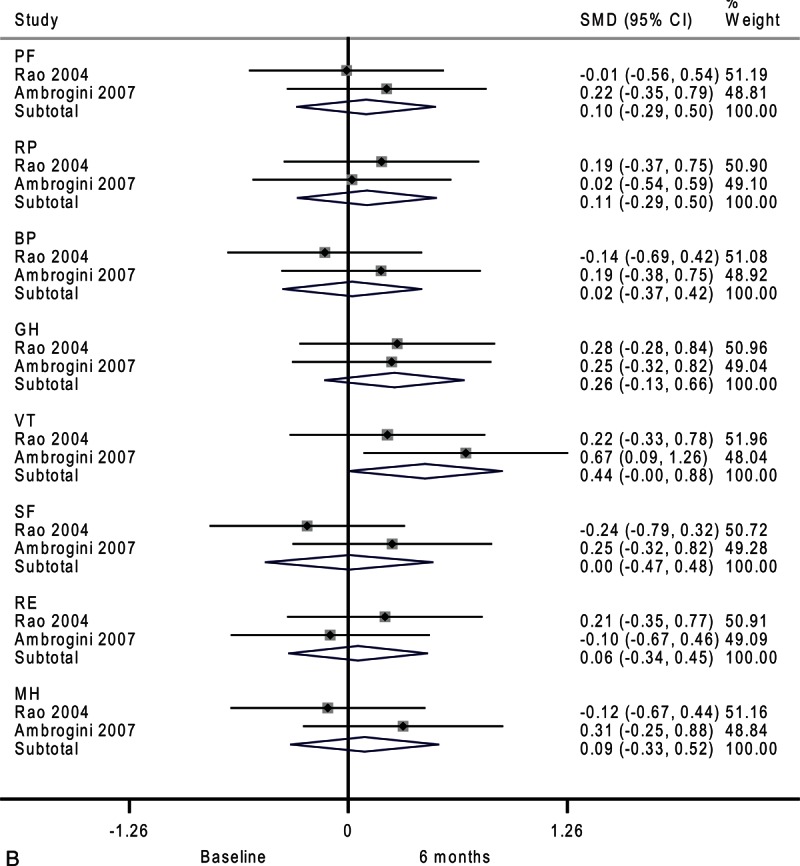
Forest plots showing standardized mean difference (SMD) and 95% confidence intervals (CIs) of the Short Form-36 (SF-36) general health survey in surveillance (A, C, E) and surgery (B, D, F) groups at 6 months (A, B), 12 months (C, D), and 24 months (E, F). The diamonds represent the overall pooled estimate.

**FIGURE 4 (Continued):**
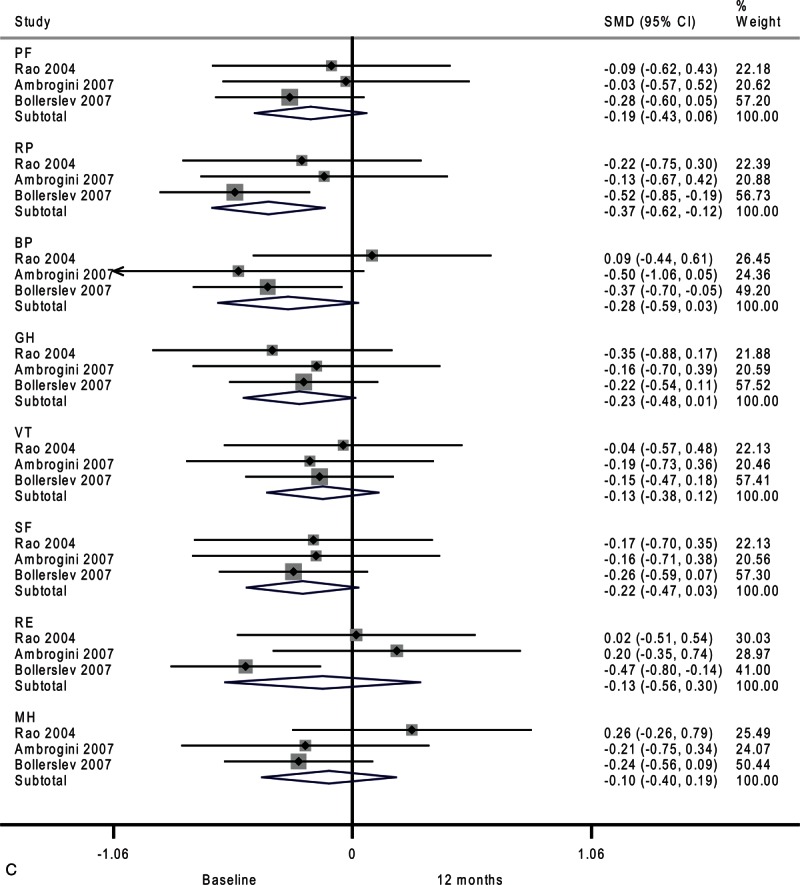
Forest plots showing standardized mean difference (SMD) and 95% confidence intervals (CIs) of the Short Form-36 (SF-36) general health survey in surveillance (A, C, E) and surgery (B, D, F) groups at 6 months (A, B), 12 months (C, D), and 24 months (E, F). The diamonds represent the overall pooled estimate.

**FIGURE 4 (Continued):**
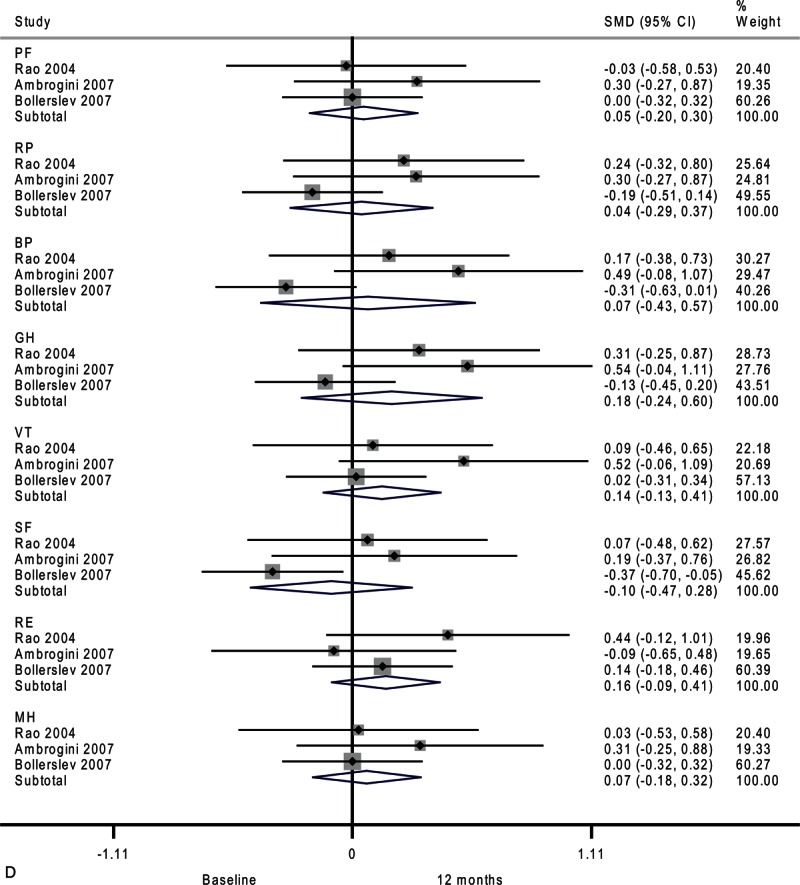
Forest plots showing standardized mean difference (SMD) and 95% confidence intervals (CIs) of the Short Form-36 (SF-36) general health survey in surveillance (A, C, E) and surgery (B, D, F) groups at 6 months (A, B), 12 months (C, D), and 24 months (E, F). The diamonds represent the overall pooled estimate.

**FIGURE 4 (Continued):**
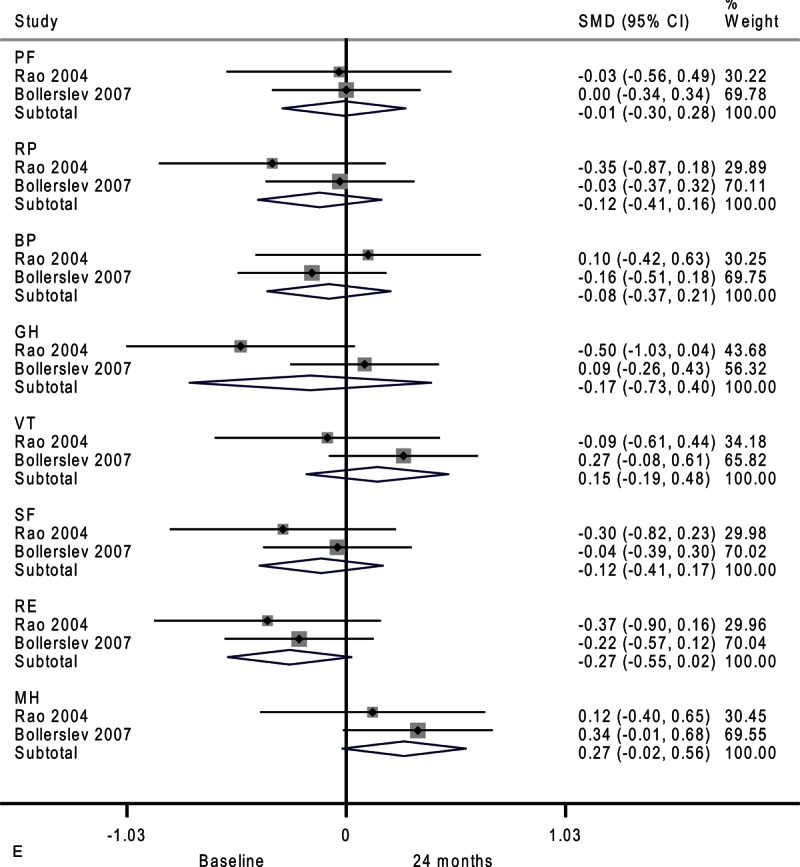
Forest plots showing standardized mean difference (SMD) and 95% confidence intervals (CIs) of the Short Form-36 (SF-36) general health survey in surveillance (A, C, E) and surgery (B, D, F) groups at 6 months (A, B), 12 months (C, D), and 24 months (E, F). The diamonds represent the overall pooled estimate.

**FIGURE 4 (Continued):**
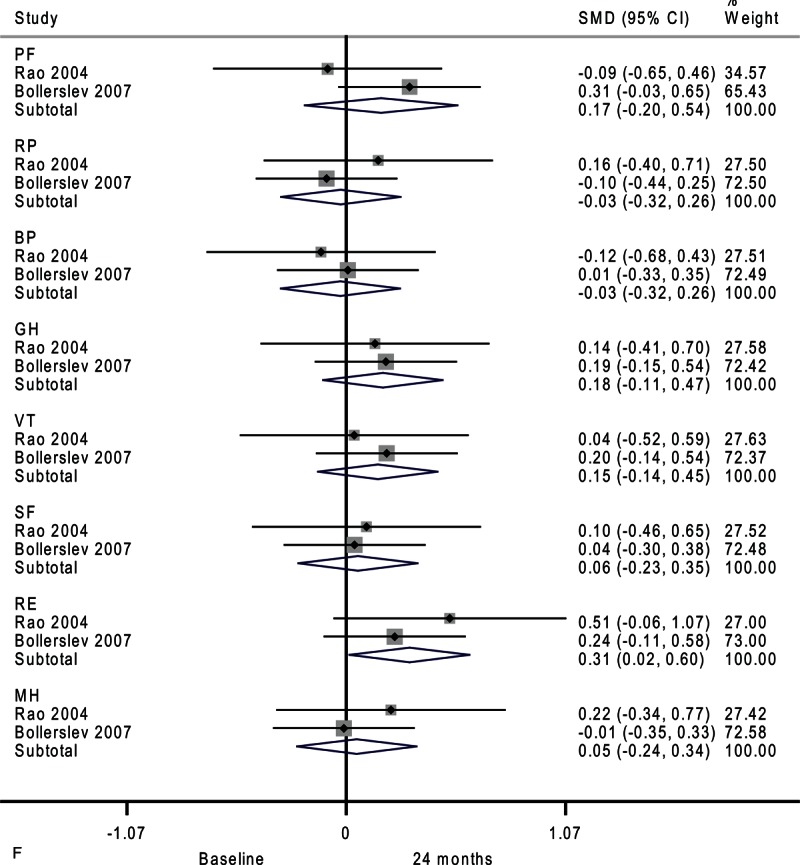
Forest plots showing standardized mean difference (SMD) and 95% confidence intervals (CIs) of the Short Form-36 (SF-36) general health survey in surveillance (A, C, E) and surgery (B, D, F) groups at 6 months (A, B), 12 months (C, D), and 24 months (E, F). The diamonds represent the overall pooled estimate.

The differences in the surveillance group disappeared after 24 months (Figure 4     E). Patients assigned to medical surveillance had worse emotional role functioning (SMD, −0.27; 95% CI −0.55 to 0.02; *P* = 0.07). Paradoxically, mental health appeared better after 24 months (SMD, 0.27; 95% CI −0.02 to 0.56; *P* = 0.07). Moderate-to-substantial heterogeneity among studies was observed in general health (*I*^2^ = 69.1%)

In the surgery group, vitality marginally improved after 6 months as shown in Figure 4     B (SMD, 0.44; 95% CI −0.00 to 0.88; *P* = 0.05). Moderate heterogeneity among studies was observed in social functioning (*I*^2^ = 30.9%).

After 12 months, the surgery group showed no significant difference across all domains of the SF-36 (Figure 4     D). There were moderate heterogeneities in physical role functioning (*I*^2^ = 33.9%), bodily pain (*I*^2^ = 69.2%), general health (*I*^2^ = 56.7%), and social functioning (*I*^2^ = 47.3%).

Compared with baseline, the surgery group had better emotional role functioning after 24 months as shown in Figure 4     F (SMD, 0.31; 95% CI 0.02–0.60; *P* = 0.04). Moderate heterogeneity among studies was detected in physical functioning (*I*^2^ = 30.7%).

## DISCUSSION

We included 3 randomized controlled trials for this meta-analysis by electronic search and manual screening. Our results suggest that emotional role functioning is consistently better in the surgery group after 24 and 48 months. Furthermore, compared with baseline, emotional role functioning improved after parathyroidectomy. The degree of heterogeneity was generally low. The present meta-analysis updates current evidence to clarify the role of surgery in patients with asymptomatic primary hyperparathyroidism.

The SF-36 emotional role functioning domain consists of the following 3 questions:

(1) cut down on the amount of time you spent on work or other activities;

(2) accomplished less than you would like; and

(3) did work or other activities less carefully than usual.

These may provide a big picture on cognitive functions. Several studies have used different instruments to characterize cognitive performance such as verbal memory and nonverbal abstraction.^15,22,23^ These cognitive functions may influence daily activities independent of depression or other mood disorders. Recently, Babinska et al^24^ showed that patients with hyperparathyroidism had impaired concentration, decreased nonverbal learning process, difficulties in using direct memory, verbal fluency, and visual constructive abilities. Only a part of aspects improved following surgery. Interestingly, Amstrup et al^25^ also demonstrated that former patients (5–15 years after parathyroidectomy) had lower scores in 3 SF-36 domains compared with sex- and age-matched healthy controls: general health, vitality, and emotional role functioning. It is unknown whether these phenomena result from irreversible effects of hyperparathyroidism or different genetic and environmental backgrounds. Therefore, observational studies using healthy or thyroidectomy controls are likely to be biased due to confounding.

Previous systematic reviews include studies of nonrandomized design and present the results in a nonquantitative manner.^14,15,22,23^ Recently, Brito et al^26^ conducted a meta-analysis of the extent of improvement in the SF-36 and Pasieka's parathyroidectomy assessment of symptoms score. The study included 4 prospective surgical series and omitted several publications, including 3 randomized controlled trials analyzed in the present study. Their results indicated significant improvement in all domains of the SF-36. Nonetheless, the findings are restricted by the relatively lower level of evidence. Perceptions about intervention effectiveness may influence treatment outcomes. Randomized controlled trials with an observational arm are necessary to get rid of the so-called Hawthorne effect or selection bias.

One of the strengths of this meta-analysis is to increase statistical power over individual studies and explore the heterogeneity between studies. Furthermore, we used a comprehensive search strategy and duplicate data abstraction to minimize bias. Our findings are limited by the small number and size of randomized controlled trials. The results need to be interpreted with caution on reliability. In addition, heterogeneity and the test for publication bias may have been underestimated because these tests are underpowered when the number of trials is few. A way to overcome this limitation is to include prospective nonrandomized studies. Two additional studies comparing surgical with medical treatment did not specify the time points of assessment of quality of life.^27,28^ Therefore, they were not incorporated in our meta-analysis.

This review is clinically relevant because integrating quality-of-life assessment is helpful in supporting clinical decision making and has potential to enhance patient-centered care. Although the SF-36 is a generic measure of health status that may not be sensitive to clinical changes specific to hyperparathyroidism, significant differences were observed between the surveillance group and surgery group in the present study. It is worth noting that our temporal analysis (Figure 4     ) revealed a trend toward worsening quality of life in the surveillance group and vice versa in the surgery group. This reinforces our conclusion that hyperparathyroidism patients undergoing surgery or surveillance present different alterations in quality of life. A well designed and adequately powered trial would be necessary to establish the magnitude and durability of improvements in quality of life after parathyroidectomy, and whether the relevant changes are related to biochemical abnormalities. Future studies should have longer follow-up to evaluate time trends to determine whether the effect is constant, linear, or prone to plateau over time.

In summary, pooled data from randomized trials suggest that quality of life significantly differs in patients with asymptomatic primary hyperparathyroidism undergoing surgery or surveillance, particularly in the domain of emotional role functioning.
